# Caries experience and oral health-related quality of life (OHRQoL) of children and adolescents with cerebral palsy in a low-resource setting

**DOI:** 10.1186/s12903-018-0704-2

**Published:** 2019-01-15

**Authors:** Rahena Akhter, Nur Mohammad Monsur Hassan, Elizabeth F. Martin, Mohammad Muhit, Hayley Smithers-sheedy, Nadia Badawi, Gulam Khandaker

**Affiliations:** 1 School of Dentistry, Faculty of Medicine and Health, The University of Sydney, C24 Westmead Hospital, Level 1 WCOH, Westmead, NSW 2145 Australia; 2 School of Dentistry and Health Sciences, Charles Sturt University, Orange, NSW Australia; 3 CSF Global, Dhaka, Bangladesh; 4 Asian Institute of Disability and Development (AIDD), University of South Asia, Dhaka, Bangladesh; 5 Cerebral Palsy Alliance Research Institute, The University of Sydney, Allambie Heights, NSW Australia; 6 The Children’s Hospital at Westmead Clinical School, The University of Sydney, Westmead, NSW Australia; 7 Public Health Unit, Central Queensland Hospital and Health Service, Rockhampton, Queensland Australia

**Keywords:** Dental caries, Cerebral palsy, Quality of life, Oral health, Children, Adolescents

## Abstract

**Background:**

Children with complex neurodevelopmental disabilities such as cerebral palsy (CP), have a higher risk of dental disease related at least in part to greater difficulties in performing and maintaining effective oral hygiene and oral care practices. However, to date, there are very few studies that have considered the impact of dental disease on the Oral Health-Related Quality of Life (OHRQoL) of children and adolescents with cerebral palsy. This study aimed to investigate the association between dental caries experience and oral health related quality of life (OHRQoL) among children and adolescents with cerebral palsy in a low-resource setting (Bangladesh).

**Methods:**

A total of 90 children and adolescents with CP, 2–17 years old (median age 10 years; 37.8% female and 62.2% male) were randomly selected from the Bangladesh Cerebral Palsy Register (BCPR) The decayed, missing and filled teeth (dmft/DMFT) index was used to measure caries experience. Child Perceptions Questionnaire (CPQ) and Family Impact Scale (FIS) were used to assess oral health–related quality of life (OHRQoL). Binary logistic regression was used to investigate factors that may contribute to dental caries experience.

**Results:**

Dental caries were observed among 55.6% of the participants. After adjusting for age and gender, binary logistic regression analysis showed that dental caries experience was significantly associated with those who had teeth/mouth pain (rate ratio 7.3; *P* = 0.02), food caught between teeth (rate ratio: 6.4; *P* = 0.02), difficulty in eating and drinking (rate ratio 5.9; *p* = 0.02) and those who felt frequently upset (rate ratio: 54.7; *P* = 0.02).

**Conclusion:**

In this study, we found that children and adolescents with CP in a low-resource setting had high dental caries experience and that dental caries had a negative impact on OHRQoL amongst these participants and their parents/caregivers. Health care professionals should be aware of the importance of dental health and oral hygiene in this population. These findings highlight the need for oral health promotion programs for children and adolescents with CP in these settings to reduce pain and to improve quality of life.

## Background

Cerebral palsy (CP) describes a group of disorders of movement and posture caused by injury or disturbances in the developing brain [1]. CP is the most common physical disability in childhood, with an estimated prevalence in high income countries of 2.1 per 1000 live births [2, 3]. Children with complex neurodevelopmental disabilities such as CP, have a higher risk of dental disease related at least in part to greater difficulties in performing and maintaining effective oral hygiene and oral care practices [4–6]. Individuals with CP can have orofacial dysfunction and parafunctional habits such as tongue thrust sialorrhea and dysphagia [7]. In our previous investigation of dental caries amongst children with CP in rural Bangladesh we found that children with more severe functional movement limitations (Gross Motor Function Classification System (GMFCS) levels IV–V) had an increased caries experience when compared to those with milder functional motor limitations (GMFCS levels I–III) [8, 9]. Untreated dental caries is often linked with discomfort, toothache, changes in body weight and growth and can have a negative impact on oral health-related quality of life (OHRQoL) of children and their families/caregivers [10]. Oral health-related quality of life (OHRQoL), as defined in the United States Surgeon General’s report on oral health, is “a multidimensional construct that broadly reflects people’s comfort when eating, sleeping, and engaging in social interaction; their self-esteem; and their satisfaction with respect to their oral health” [11].

Several studies have examined the OHRQoL in individuals with CP [4, 5, 12]. A Brazilian study of children living with CP [4] found that severity of dental caries and low family income were both strongly associated with a negative effect on OHRQoL. A study from Hong Kong compared the health quality of life (HQoL) and oral health quality of life (OHRQoL) between preschoolers with and without CP and found that the HQoL and OHQoL were significantly lower in children with CP [12]. However, most studies to date have reported on highly selected groups of children with CP, such as those who attended specialized clinics or rehabilitation centers. Although CP is estimated to be considerably more common in resource-poor settings [8, 13–15], to the best of our knowledge, there have been no studies investigating the OHRQoL of children and adolescents with CP in low- and middle-income countries (LMICs). Understanding OHRQoL in LMIC settings is necessary to inform policy and oral health care programs for people with CP. In this study we investigated the caries experience and its impact on OHRQoL amongst children and adolescents with CP in a low resource setting (Bangladesh).

## Methods

### Study population and data collection

The sampling frame for this study was from the Bangladesh CP Register’s (BCPR) program of research. In a LMIC setting, the BCPR is the first population-based CP register and has been operating since January 2015 [15]. We used systematic random sampling to select a sub-group of 90 children and adolescents with CP aged less than 18 years from BCPR program for our oral health component of the study. The recruitment strategies and characteristics of the study population have been described previously [9, 16]. The BCPR only includes children/adolescents with a clinical description of CP [17]. Each record includes details such as CP motor type (spastic, dyskinetic, ataxic and hypotonic), the spastic subtype (mono/hemiplegia, diplegia, triplegia and quadriplegia) and functional gross motor abilities (GMFCS) [8, 9].

### Children’s oral examination

The oral health assessment included a calibration process that had two stages: a theoretical stage and a clinical stage and have been described previously [9]. Following this calibration all participants (children and adolescents with CP) were examined by the trained dentist in a local primary school or an NGO centre with the patient sitting on a traditional chair [9]. To conduct the oral examination, the dentist used: a 250-lm LED (Light Emitting Diode) light bulb coupled to the head, flat dental mirrors, mouth openers, wooden spatulas and disposable gauze [18, 19].

Caries was assessed according to the 2013 World Health Organization (WHO) criteria for dental caries to identify decayed, missing and filled teeth (dmft) for deciduous teeth and Decayed, Missing and Filled teeth (DMFT) for permanent teeth indices. Dmft/DMFT caries prevalence was numerically expressed by calculation of the number of carious, missing and filled teeth for each individual. The overall values of dmft and DMFT were evaluated separately and together by the sum of d + m + f + D + M + F. The scores were given, and the severity of dental caries was expressed based on dmft+DMFT = 0 caries free and dmft+DMFT> 0 presence of caries. If a retained deciduous tooth was present, the caries status of only the permanent teeth were recorded, as per WHO guidelines [9].

### OHRQoL instrument

A 24-item questionnaire combining questions from the Child Perceptions Questionnaire (CPQ) and the Family Impact Scale (FIS) were used as the OHRQoL measurements in this study [5]. The CPQ was used to obtain data from the primary caregivers or parents as many of the children and adolescents were unable to self-report. The questions referenced the frequency of events in the previous three months. The questionnaire consisted of 2 sections, the first section was marked as “yes” or “no” and the second section was scored using a four-point Likert scale (response options: sometimes = 1, often = 2, everyday = 3, and almost every day = 4).

The first section titled, “Questions in the CPQ (Child Perceptions Questionnaire),” consisted of 16 questions where parents were asked to rate the effect of their child’s current oral health on their daily lives (i.e. “how often have you had mouth sores because of your teeth/mouth?”). The items in this section were: pain in teeth/mouth, bad breath, mouth sores, food caught between teeth, difficulty eating/drinking hot/cold foods, difficulty chewing firm foods, difficulty saying words, taking longer to eat a meal, trouble sleeping, felt upset, felt irritable/frustrated, felt shy, concerned what people think about your teeth/mouth, teased/called names, avoided smiling/laughing, argued with children/family and not wanted to speak/read loud in class [4, 20].

The second section titled, “Questions in the FIS, Family Impact Scale,” contained 8 questions where parents were asked to rate their concerns about their child’s oral health (eg., “how often have you had disrupted sleep because of your child’s teeth/mouth?”). The items in this section were: felt guilty, been upset, had disrupted sleep, required more attention from you or others in the family, taken time off work, had less time for yourself or the family, blamed you or another person in the family, and argued with you or others in the family [5].

### Statistical analysis

The data were processed using SPSS (Statistics Package for Social Science, version 22.0 for windows; SPSS Inc., Chicago, IL USA). The measures of central tendency (mean) and dispersion (SD) were used for DMFT and dmft to characterize each participant’s caries experience. The association between caries experience (dmft+DMFT> 0) and the OHRQOL variables as CPQ and FIS were assessed using X^2^ tests. Binary logistic regression analysis was used and adjusted by age and sex. Odds ratios (ORs) and 95% confidence intervals (CIs) were calculated from the model. The significance level was set at *p* < 0.05.

## Results

A total of 90 children with CP were enrolled in this study (mean age 9y 7mo, range 2–17y), of whom 37.8% were female and 62.2% were male. According to the spastic motor type 36.7% of children and adolescents were diagnosed with mono/hemiplegia, 17.8% with diplegia, 21.1 tri/quadriplegia and 24.4% were diagnosed as dyskinetic, ataxic or hypotonic. GMFCS classification were 37.8, 18.9, 17.8, 7.8 and 17.8% (GMFCS I-V, respectively). The average deciduous teeth and permanent teeth caries experience scores (dmft and DMFT) were 2.46 (3.75) and 0.72 (1.79) respectively. Mean (standard deviation [SD]) values of the combined caries experience (dmft+DMFT) were 3.18 (4.58). This data has previously been reported by Akhter et al., 2017 [9].

Dental caries experience exerted a significant negative impact on OHRQoL, especially for participants who had tooth/mouth pain (*p* = 0.001), bad breath (*p* = 0.03), trouble sleeping (*p* = 0.03) or avoided smiling (*p* = 0.02) (Table 2). Using X^2^ tests, we found statistically significant differences (*p* = 0.0001) in mealtime experience amongst participants with caries compared to those without. Specifically, this pertained to the proportion who reported having food caught between teeth and difficulty in eating or drinking (Table 1). The main concerns for parents of children and adolescents with CP were that they felt guilty and upset.

**Table 1:** Percentage of CP children and adolescents who are caries free and caries present according to the CPQ variables

CPQ variables	Total number of children and adolescents with CP, *n* = 90	
	Caries free, *n* = 43 (%)	Caries present, *n* = 47 (%)
Pain in teeth/mouth		
No	55.6	44.4
Yes	18.5	81.5***
Bad Breath		
No	55.6	44.4
Yes	33.3	66.7*
Mouth sores		
No	43.7	56.3
Yes	66.7	33.3
Food caught between teeth		
No	60.7	39.3
Yes	10.3	89.7***
Difficulty in eating, drinking or chewing firm foods		
No	63.6	36.4
Yes	14.3	85.7***
Difficulty in saying words		
No	46.7	53.3
Yes	43.3	56.7
Taken longer to eat a meal		
No	47.9	52.1
Yes	31.6	68.4
Trouble sleeping		
No	50	50
Yes	22.2	77.8*
Upset		
No	43.5	56.5
Yes	46.4	53.6
Felt terrible or frustrated		
No	45.8	54.2
Yes	41.8	58.1
Felt shy		
No	37.7	62.3
Yes	58.6	41.4
Concerned what people think about your teeth/mouth		
No	43.7	56.3
Yes	66.7	33.3
Teased/called names		
No	43.3	56.6
Yes	50	50
Avoid smiling or laughing		
No	51.5	48.5
Yes	22.7	77.3*
Argues with children or family		
No	44.2	55.8
Yes	50	50
Not wanted to speak or real loud in class		
No	47.3	52.7
Yes	40	60

*P < 0.05 and ***P < 0.001, Significance tested by Chi-square test

Table 2 demonstrates significant associations with dental caries experience and FIS parents reports of ‘ felt frequently guilty’ (*p* = 0.001) and ‘being upset’ (*p* = 0.0001).

**Table 2:** Percentage of children and adolescents with CP by caries status and FIS variables

FIS variables (Because of their teeth/mouth)	Total number of children and adolescent with CP, *n* = 90	
	Caries free, *n* = 43 (%)	Caries present, *n* = 47 (%)
Felt guilty		
No	68.2	31.8
Yes	36.8	63.2**
Been upset		
No	77.4	22.6
Yes	27.1	72.9***
Had disrupted sleep		
No	43.8	56.2
Yes	47.1	52.9
Required more attention from you or others in the family		
No	45.5	54.5
Yes	42.9	57.1
Taken time off work		
No	45.9	54.1
Yes	20	80
Had less time for yourself or the family		
No	47.6	52.4
Yes	37	63
Blamed you or another person in the family		
No	44.9	55.1
Yes	41.7	58.3
Argues with you or others in the family		
No	43	57
Yes	75	25

***P < 0.001 and **P < 0.01, Significance tested by Chi-square test

After adjusting for age and gender, binary logistic regression analysis showed that dental caries experience was significantly associated with CPQ and FIS scores among children and adolescents with CP; especially in those children and adolescents who reported feeling upset frequently (*p* = 0.02).

Amongst children and adolescents with CP who had caries present, pain in the teeth/mouth was approximately 7.3-times more prevalent, food caught between teeth was approximately 6.4-times higher, and having trouble in drinking, eating or chewing firm foods was approximately 5.9-times greater than for children and adolescents without caries experience (Table 3).

**Table 3:** Multivariate assessment of the association between the prevalence of dental caries and independent variables

Variables (comparison groups)	Total number of children and adolescents with CP, *n* = 90	
	Odds ratio^a^ (95% CI)	*P*-value
Child perceptions questionnaire		
Pain in teeth/mouth		
No	Reference group	
Yes	7.27 (1.38–38.37)	0.01
Food caught between teeth		
No	Reference group	
Yes	6.37 (1.32–30.81)	0.02
Difficulty in drinking, eating or chewing firm foods		
No	Reference group	
Yes	5.89 (1.31–26.16)	0.02
Family Impact Scale		
Been upset because of teeth/mouth		
No	Reference group	
Yes	54.7 (2.05–1457.73)	0.01

^a^Prevalence ratio and 95% confidence interval, adjusted by age and gender, as assessed by binary logistic regression analysis, Reference group: Caries present, dmft+DMFT> 0

## Discussion

This study evaluated the impact of dental caries experience and its impact on the oral health related quality of life in children and adolescents with CP in rural Bangladesh. To the best of our knowledge, this is first study showing the association of dental caries on the daily functioning, well-being and oral health related quality of life in children and adolescents with CP in a low-resource setting. This study highlights the significant role that dental caries has on OHRQoL amongst children and adolescents with CP.

In this study we found the mean of combined caries experience (dmft+DMFT) of children and adolescents living with CP in Bangladesh was 3.18. Previous research from Bangladesh reported that children aged 6 years without CP had an average caries experience score of 1.1 and children and adolescents aged 8 to 12 years had a score of 1.72, this is significantly less than the mean found in this study of children and adolescents living with CP [21, 22]. The high caries experience and subsequent negative impact on OHRQoL reported in our findings highlights the importance of oral health amongst children and adolescents with CP. These findings may represent the more severe end of the spectrum of OHRQoL amongst children and adolescents with CP as this sample was drawn from a rural area, consisting of a poorer socio-economic group (average monthly family income <US$70) where most of the participants had no history of visiting a dentist or dental clinic.

Parental perceptions of dental caries experience and pain in the teeth and mouth, getting food caught between teeth, difficulty eating and drinking and feeling upset frequently were all strongly associated with a negative impact on the OHRQoL of children and adolescents with CP. As children with CP who have more severe functional mobility limitations (GMFCS IV and V) have greater caries experience [9] these children are likely to be at high risk for poor OHRQoL. Our findings are also in line with previous studies which reported that refusal of food was an important issue amongst children with CP who had dental caries experience [5].

Parents and caregivers are indispensable members of the multi-disciplinary team as they provide daily support for children and adolescents living with CP. Their completion of the CPQ and FIS representing the children and adolescents with CP provided important information particularly in relation to whether quality of life factors were associated with pain in their child’s teeth/mouth. Previous studies have reported that parents of children with CP demonstrate higher levels of distress due to their children’s oral health than parents of children without CP. El Ashiry et al. indicated that parents of children with CP had greater uneasiness regarding their child’s oral health than those parents of children without CP, and a study in Hong Kong found the same association with parents of preschool children with CP [4, 5, 12]. Parents and caregivers of children with CP may be more likely to experience uneasiness or difficulties supporting their children’s daily oral hygiene activities due to complications related to intraoral sensibility, presence of involuntary physical movements and/or oral pathological reflexes and spasticity in masticatory muscles [23] common to many children with CP.

## Study limitations

There are several limitations in our study. Parents acted as proxies for children and adolescents in this study and as such the results may not fully reflect the children and adolescents feelings and experiences. Moreover, some findings reported by caregivers might reflect factors relating to their child’s cerebral palsy rather than the dental condition (DMFT) alone. Our study was also based on a small sample. However, we selected a random sample of children and adolescents drawn from a population-based CP register reducing any potential selection bias and providing the first published research describing the oral health quality of life of children and adolescents in LMIC in rural Bangladesh. This information will further emphasize efforts towards caries prevention, screening and early detection of oral symptoms and problems.

## Conclusion

Our data suggests that children and adolescents with CP who have dental caries are at increased risk of negative oral health related quality of life. Efforts should be made to develop an effective oral health promotion program for children and adolescents with CP in rural Bangladesh and other resource scarce settings.

## Abbreviations

BCPR: Bangladesh Cerebral Palsy Register
CP: Cerebral palsy
dmft: Decayed, missing, and filled teeth for deciduous teeth
DMFT: Decayed, Missing, and Filled teeth for permanent teeth
LMIC: Low- and middle-income countries
